# Spinal Cord Infarction in a Patient with Hereditary Spherocytosis: A Case Report and Discussion

**DOI:** 10.1155/2016/7024120

**Published:** 2016-03-14

**Authors:** Waqar Waheed, Anjali L. Varigonda, Chris E. Holmes, Christopher Trevino, Neil M. Borden, W. Pendlebury

**Affiliations:** ^1^Department of Neurological Sciences, University of Vermont College of Medicine, Burlington, VT 05401, USA; ^2^Department of Psychiatry, University of Vermont College of Medicine, Burlington, VT 05401, USA; ^3^Hematology/Oncology Division, Department of Medicine, University of Vermont College of Medicine, Burlington, VT 05401, USA; ^4^Department of Radiology, University of Vermont College of Medicine, Burlington, VT 05401, USA

## Abstract

The etiology of spinal cord infarcts (SCIs), besides being related to aortic perioperative events, in large subset of SCIs, remains cryptogenic. We present a first case of SCI in a patient with hereditary spherocytosis and discuss the potential pathophysiologic considerations for vascular compromise. A 43-year-old woman with a history of hereditary spherocytosis, post splenectomy status, presented with chest, back, and shoulder pain with subsequent myelopathic picture; SCI extending from C4-T2 was confirmed by MRI. Despite aggressive treatment her stroke progressed leading to her demise. Her autopsy confirmed the SCI and revealed some incidental findings, but the cause of SCI remained unidentified. Exclusion of the known etiologies of SCI by extensive negative workup including autopsy evaluation suggested that SCI in our case was related to her history of hereditary spherocytosis. Both venous and arterial adverse vascular events, at a higher rate, have been associated in patients with hereditary spherocytosis who had their spleens removed compared to nonsplenectomized patients. Postsplenectomy increases in the platelet, red blood cell count, leukocyte count, and cholesterol concentrations are postulated to contribute to increased thrombotic risk. Additional prothrombotic factors include continuous platelet activation and adhesion as well as abnormalities of the red blood cell membrane.

## 1. Introduction

Spinal cord infarcts (SCIs) are very uncommon events that carry immense morbidity and mortality. SCIs encompass less than 1% of all strokes and 5–8% of acute myelopathies [1]. Although the majority of SCIs are associated with perioperative procedures (mostly aortic surgeries) [2], there remains a large subset of SCIs of uncharacterized etiology. This case report serves to shed light on a case of spinal cord infarction in a patient who had hereditary spherocytosis, for which she had undergone splenectomy during childhood. To our knowledge, the example of a spinal cord infarction as a deleterious vascular event in the setting of hereditary spherocytosis has not been previously reported. We will discuss the potential pathophysiologic and clinical considerations of this case.

## 2. Case Presentation

A 43-year-old woman presented to the emergency department with acute onset of chest, back, and bilateral shoulder and upper arm pain, which started upon rising from a seated position. Subsequent cardiac workup was negative and the pain resolved with ibuprofen, and she was admitted for observation. Over the course of the following day the patient developed bilateral numbness and weakness in her lower extremities, which started sequentially with the right leg progressing to the left leg, and, by the following day, had involvement of the bilateral arms. There was no associated trauma; review of systems was negative for constitutional, cardiopulmonary, or abdominal symptoms.

Past medical and surgical histories were significant for hereditary spherocytosis, morbid obesity, hypertension, primary hyperparathyroidism with associated hypercalcemia and nephrolithiasis, postparathyroidectomy status, borderline diabetes mellitus, mild intellectual disability, and depression. She underwent splenectomy at the age of 14 years for hereditary spherocytosis. She had hematologic follow-up a decade after her splenectomy for persistent leukocytosis and thrombocytosis, attributed to lack of splenic sequestration, further suggested by negative JAK2 and BCR-ABL mutation. About 2 years prior to presentation, she had an unprovoked superficial venous thrombosis of her right lower extremity, for which no hypercoagulability workup was completed.

Home medications included cholecalciferol (Vitamin D3) 2000 IU/day, citalopram 20 mg/day, and lisinopril 10 mg/day. She was a nonsmoker and nonalcoholic with no history of illicit drug use. There was no family history of bleeding or thrombosis disorders.

Her admitting vitals showed pulse of 67/minute, blood pressure of 117/67, and temperature of 36.5°C. Her initial examination in ER showed variable fluctuating weakness and inconsistent sensory findings, confounded by her baseline history of developmental delay; however, subsequent neurologic examination was notable for a myelopathic pattern of weakness, suggested by quadriparesis, legs > arms, hyperreflexia in arms, absent reflexes in legs, loss of pain and temperature in lower extremities with sensory level at T5-6 level, spared proprioception, and vibration as well as loss of sphincter control. Bilateral Babinski signs were negative.

## 3. Laboratory Data

**Figure 1:**
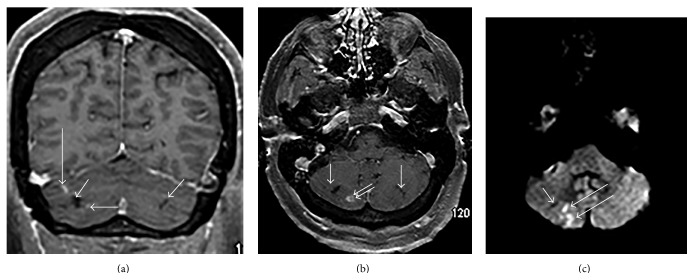
(a) Coronal postcontrast T1 weighted magnetic resonance (MR) image shows areas of old (chronic) infarction in the equators of both cerebellar hemispheres (short white arrows) and small, enhancing subacute infarcts in right cerebellum (long white arrows). (b) Axial postcontrast T1 weighted MR image shows areas of old (chronic) infarction in both cerebellar hemispheres (single white arrows) and an enhancing subacute infarct in the right cerebellum (double white arrow). (c) Axial diffusion weighted MR image (DWI) through the posterior fossa shows a small, old right cerebellar infarct (short white arrow) and areas of subacute infarction with diffusion restriction (long white arrows).

**Figure 2:**
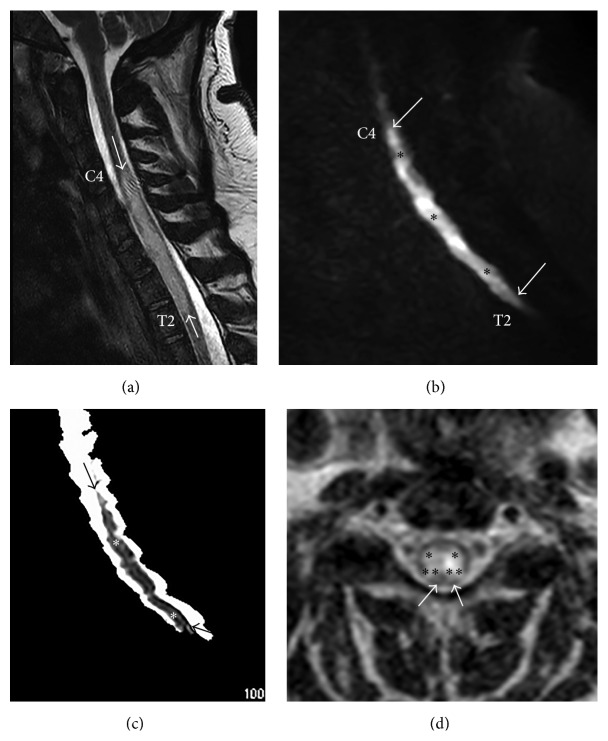
(a) Sagittal T2 weighted Fast Spin echo (FSE) MR image shows spinal cord expansion (swelling) with abnormal increased T2 signal extending from the C4 level to the T2 level (between the white arrows). (b) Sagittal DWI trace image shows abnormal increased signal intensity between C4 and T2, corresponding to the abnormal T2 signal noted on Sagittal T2 FSE imaging. (c) Sagittal apparent diffusion coefficient (ADC) map shows abnormal restriction of diffusion (dark signal in spinal cord indicated by arrows and asterisks) indicating that the increased signal on the DWI trace image represents diffusion restriction (not T2 shine through). (d) Axial T2 weighted FSE image at the C4 level shows cord expansion (swelling) and abnormal increased T2 signal within the swollen, edematous central gray matter (single asterisks indicate anterior horns and double asterisks indicate posterior horns of central gray matter). White arrows indicate uninvolved dorsal columns of the spinal cord.

Magnetic resonance imaging (MRI) of the brain obtained at the time of initial presentation demonstrated areas of old, chronic infarction at the equators of both cerebellar hemispheres (short white arrows in Figures 1(a) and 1(b)), in the watershed regions between the superior cerebellar and posterior inferior cerebellar artery vascular territories. There were also enhancing areas of subacute infarction in the right cerebellum (long white arrows in Figures 1(a), 1(b), and 1(c)). MRI of the cervical and thoracic spine obtained at this time revealed expansion of the cervical and upper thoracic spinal cord with abnormal increased T2 signal (between the white arrows in Figure 2(a)) with prominent diffusion restriction (between arrows in Figures 2(b) and 2(c)) extending from the inferior C4 level to the mid T2 level. The cord signal abnormality was primarily confined to the central gray matter (asterisks in Figure 2(d)). The clinical presentation and imaging findings were most compatible with anterior spinal artery distribution acute SCI; however, considering the possible patchy enhancement, transverse myelitis remained initially on the differential diagnosis. Lumbar puncture revealed normal results (normal cell count, protein, gram stain, cultures, oligoclonal banding IgG index, and cytology). CT angiogram of her neck and cervical spine was normal with no evidence of dissections or stenosis. A cardioembolic source was excluded with a sinus rhythm EKG and transthoracic echocardiogram demonstrating only mild left ventricular hypertrophy.

Abnormal labs included a complete blood count which showed baseline leukocytosis (34.3, normal 4–12 K/cmm) and thrombocytosis (486, normal 141–320 K/cmm), Hemoglobin A1c (6.3, normal 5.7–6.4%), and C reactive protein (13.9, normal < 1.0 mg/dL). Complete metabolic profile, TSH, Vitamin B12, folic acid, PT, PTT, and urine toxicology screen were normal. Vasculitis markers (ESR, ANA, anti-ds DNA, anti-SSA/SSB and ANCA), anti-NMO, and paraneoplastic panel (Mayo Clinic, Rochester, Minnesota, USA) were normal. Her lipid profile revealed cholesterol of 176 mg/dL, LDL of 108 mg/dL, HDL of 43 mg/dL, and triglycerides of 125 mg/dL.

## 4. Treatment

The patient was started on Aspirin 325 mg/day, atorvastatin 80 mg/day, and subcutaneous enoxaparin deep venous thrombosis prophylaxis dose. Methylprednisolone 1 g intravenous once daily was given for 3 days while the patient was evaluated for a demyelinating disorder.

## 5. Hospital Course

On second hospitalization day, she was found to be hypotensive (86/54) with low urine output, attributed to low PO input and dehydration; discontinuation of lisinopril and intravenous fluid administration normalized her blood pressure throughout the remainder of her hospital stay.

On the sixth day of hospitalization, the patient was transferred to ICU for difficulty with clearing secretions and respiratory distress, prompting Chest CT which showed multiple bilateral pulmonary emboli, while venous Doppler ultrasound of the lower extremities was negative for deep venous thrombosis. The patient was started on full dose of anticoagulation with a heparin drip and, after obtaining hematology consultation, folic acid 1 mg for spherocytosis and hypercoagulable workup initiated. Subsequently IgG and IgM anticardiolipin antibodies, anti-beta2-glycoprotein 1 antibodies, dilute Russell's viper venom time, factor V Leiden R506Q mutation by PCR, prothrombin G20210A gene mutation by PCR, PI linked antigen, antithrombin III activity, and protein C activity and protein S activity were within normal limits.

**Figure 3:**
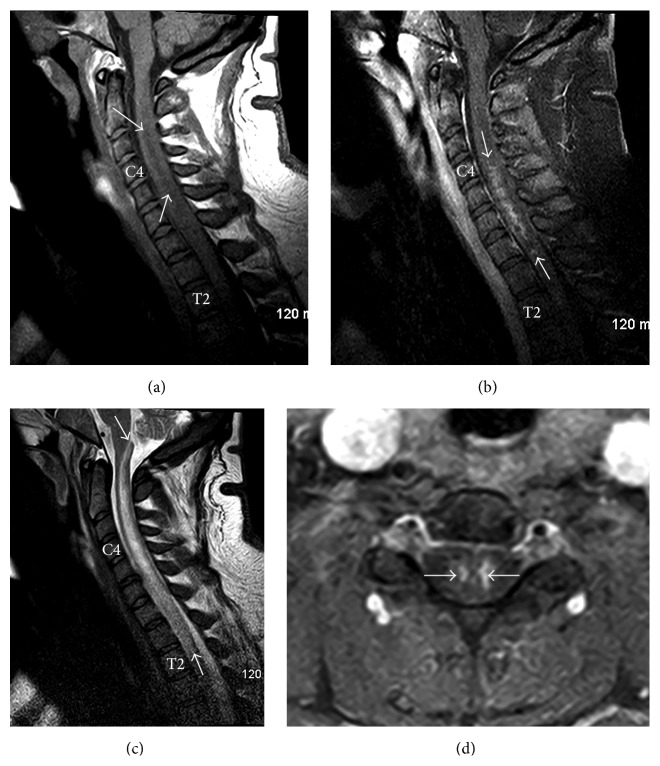
(a) Sagittal T1 weighted FSE image shows superior extension of the spinal cord swelling and abnormal decreased signal intensity centrally (between the white arrows). Note normal signal intensity of the osseous vertebral segments. (b) Sagittal T2 weighted FSE image shows superior extension of the spinal cord swelling and abnormal increased signal intensity centrally (between the white arrows) which now extends to the level of the obex. Note normal signal intensity of the osseous vertebral segments. (c) Sagittal postcontrast T1 weighted FSE image shows patchy enhancement within the central portions of the spinal cord (between the white arrows). (d) Axial postcontrast T1 weighted FSE image at the C4 level shows patchy enhancement confined to the central gray matter of the spinal cord, indicated by the white arrows (more prominent on the viewers right).

Despite maximal supportive treatment, the patient continued to progress with repeat imaging obtained 6 days after initial presentation revealed extension of the spinal cord expansion and cord signal abnormality (decreased T1 in Figure 3(a) and increased T2 in Figure 3(b)) which now extended superiorly to the level of the obex, compatible with progressive, superior extension of the spinal cord infarction. Postcontrast imaging revealed patchy areas of enhancement confined to the central gray matter (Figures 3(c) and 3(d)).

**Figure 4:**
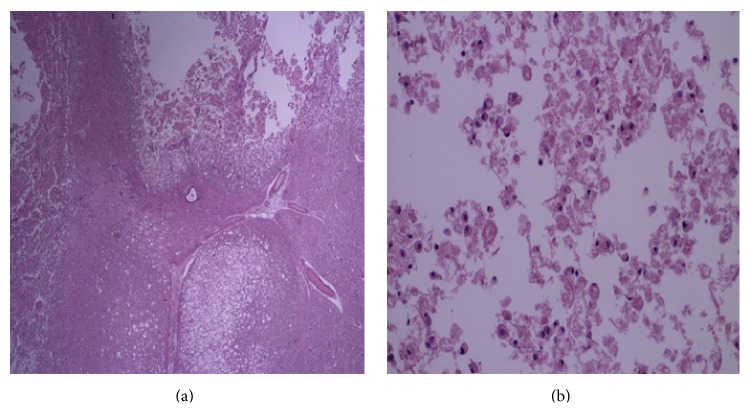
Low power of the central cervical cord (note central canal) showing cystic areas of acute infarct; 2xb is a low power of the lateral cervical cord with similar changes; high power showing a cystic area with debris and macrophages.

A family meeting was held to discuss the likelihood that she would require intubation and would likely require a tracheostomy and ventilator. She ultimately decided to pursue comfort measures instead of intubation and passed away within 24 hours. On autopsy, besides infarction of the anterior two-thirds of the cervical and upper thoracic spinal cord (Figures 4(a) and 4(b)) and left cerebellum, additional findings included two small neuroendocrine carcinoid tumors involving right lung, dilated and hypertrophic cardiomyopathy, two cystic masses of the right kidney, and a ductal plate abnormality involving the liver.

## 6. Discussion

Our case highlights some known facts of a spinal cord stroke [1–5]. (a) Unlike cerebral infarction, which is usually not painful, most (>80%) spinal infarcts are painful and, like our patient, due to involvement of afferent visceral pathways from the cardiac plexus can be mistaken for cardiac ischemia. (b) The most common clinical presentation (in approximately 95%) of SCI is an anterior spinal artery syndrome with neurologic dysfunction arising from a lesion located in the anterior two-thirds of the spinal cord. Typically, there is involvement of the central gray matter and adjacent white matter tracts, including the corticospinal, lateral spinothalamic, and autonomic tracts. Infarction with increased T2 signal of the anterior horn of the central gray matter can result in the “owl's eyes” sign described on MRI in anterior spinal artery infarction, though this imaging finding is nonspecific and has been described with other etiologies including infection (poliomyelitis), inflammation (acute idiopathic transverse myelitis), chronic compression myelopathy, and traumatic cord contusion. (c) Depending on the level and territory of spinal cord infarct, patients may develop respiratory distress; in our case C3 to C5 involvement through impairment of diaphragm and the accessory muscles of ventilation, coupled with atelectasis, pulmonary embolism, and caudal brainstem involvement, contributed to respiratory failure. (d) Hemodynamic instability owing to a greater splanchnic nerve palsy (T4–T9 lesion), requiring supportive therapy, can be seen. (e) Imaging of acute SCI related to the anterior spinal artery vascular distribution most often consists of spinal cord swelling (expansion) with abnormal decreased T1 and increased T2 signal involving the central gray matter and adjacent white matter tracts with diffusion restriction. The presence of vertebral body infarction (seen in 4–35% of patients) can provide confirmatory evidence of spinal cord infarction. In the subacute setting patchy contrast enhancement is often present. The main differential consideration for acute spinal cord infarction is transverse myelitis which typically does not demonstrate diffusion restriction and is not associated with vertebral body infarction.

SCI is much less common than cerebral infarction. The possible etiologies include atheromatosis, embolization (cardiogenic, fibrocartilaginous), severe hypotension, pathologic lesions in the aorta (aortic dissection, atherosclerosis, and aortic surgery), venous occlusion, vascular malformation, vasculitis, decompression sickness, vascular neoplasms of the spine (hemangioblastomas and cavernous angiomas), iatrogenicity, and trauma [1, 4, 5]. These etiologies were excluded by appropriate investigations and further verified by autopsy.

Fibrocartilaginous embolism, related to herniation of intervertebral disc material into the spinal vasculature, was suspected clinically based upon severe pain at the onset, a symptom-free interval between the onset of pain and deficits, and evolution of deficits over 15 minutes to 48 hours, which suggests a “spinal stroke in evolution.” However, this was excluded by lack of radiological features [6] (prolapsed disk space at the appropriate level or intersomatic disk collapse, Schmorl's nodules) and by lack of pathological evidence of emboli in the spinal vessels on autopsy. Similarly cardioembolic, aortic, and paradoxical embolizations were excluded by normal cardiac echo, telemetry, and CTA head and neck and were further ruled out by autopsy.

The presence of ductal plate abnormalities in the liver and vascular anomalies in the kidney on autopsy suggested that a vascular malformation might have been the cause of our patient's SCI, which through mechanisms of venous hypertension can lead to cord edema and eventually infarction. However, the typical clinical presentation for venous infarction is a gradually progressive myelopathy and MRI features of perimedullary flow voids were absent in our case [7]. The two small bronchial neuroendocrine tumors (NET) identified on autopsy most probably represented an incidental finding due to the lack of associated systemic features and absence of prolonged severe hypotension, which makes NET associated carcinoid crisis an untenable diagnosis [8].

The extensive negative evaluation including autopsy for SCI including hypercoagulable, vasculitis, autoimmune, neoplastic, paraneoplastic, and cardioembolic/aortospinal vasculature workup suggested that a major consideration in the etiology of spinal cord as well as subacute and chronic watershed cerebellar infarcts and pulmonary embolism in our patient was her history of hereditary spherocytosis. Splenectomy, a standard procedure for many hematological conditions including myeloproliferative disorders, hereditary stomatocytosis, thalassemia, sickle cell disease, and sideroblastic anemia, has been associated with postsplenectomy thrombocytosis and increased risk of delayed vascular complications [8–16]. Even though thromboembolic complications have most frequently been reported in thalassemia [17], there have been an increasing body of evidence linking delayed thromboembolic events involving both venous and arterial circulation with hereditary spherocytosis [4, 15–17]. The cumulative incidence of adverse arterial and venous events by the age of 70 was significantly higher in patients with hereditary spherocytosis who had their spleens removed compared to nonsplenectomized patients [18]. In another study, the reported rate of arterial events after age of 40 years was approximately five times higher in hereditary spherocytosis patients without a spleen than in hereditary spherocytosis patients with a spleen. Most of the deaths associated with these events occurred more than fifteen years after splenectomy [19].

Delayed adverse vascular events previously reported in association with postsplenectomy hereditary spherocytosis patients include myocardial infarction, cerebral infarction, pulmonary hypertension, portal vein thrombosis, pulmonary embolism, peripheral venous thrombosis, thrombotic thrombocytopenic purpura, and priapism [9–12, 14–17, 20–24]. Progressive myelopathy due to extramedullary hematopoiesis has also been described in hereditary spherocytosis; it was not confirmed on the neuroimaging studies in our case [13].

The pathophysiology that underpins the increased thrombosis risk in patients with hereditary spherocytosis is not fully elucidated [25]. Postsplenectomy increases in the platelet, hemoglobin, red blood cell count, leukocyte count, C-reactive protein levels, and cholesterol concentrations are postulated to contribute to increased thrombotic risk after splenectomy [25–27]. Continuous platelet activation and adhesion, combined with abnormalities of the red blood cell membrane through increased viscosity and sludging of abnormal red blood cells, may also play a role in increased thrombotic risk [28]. Additional hypercoagulable mechanisms such as increased thrombin generation, a decrease in natural anticoagulants, and an increase in phospholipids such as phosphatidylserine on the abnormal red blood cells have been documented in other congenital anemias but have not been clearly elucidated in patients with hereditary spherocytosis [29, 30].

In conclusion, our case expands the spectrum of adverse vascular events after splenectomy in hereditary spherocytosis and supports an association between splenectomy and increased risk of thrombotic events. Delayed ischemic events in splenectomized patients with hereditary spherocytosis, although not commonly reported, are likely underappreciated. SCI could be a hitherto unreported complication seen after splenectomy in hereditary spherocytosis. Further research is needed to elucidate the pathophysiological mechanisms behind delayed thromboembolic complications in patients with hereditary spherocytosis requiring splenectomy.

## References

[1] Rubin M. N., Rabinstein A. A. (2013). Vascular diseases of the spinal cord. *Neurologic Clinics*.

[2] Robertson C. E., Brown R. D., Wijdicks E. F. M., Rabinstein A. A. (2012). Recovery after spinal cord infarcts: long-term outcome in 115 patients. *Neurology*.

[3] Faig J., Busse O., Salbeck R. (1998). Vertebral body infarction as a confirmatory sign of spinal cord ischemic stroke: report of three cases and review of the literature. *Stroke*.

[4] Masson C., Pruvo J. P., Meder J. F. (2004). Spinal cord infarction: clinical and magnetic resonance imaging findings and short term outcome. *Journal of Neurology, Neurosurgery and Psychiatry*.

[5] Rabinstein A. A. (2015). Vascular myelopathies. *CONTINUUM Lifelong Learning in Neurology*.

[6] Duprez T. P., Danvoye L., Hernalsteen D., Cosnard G., Sindic C. J., Godfraind C. (2005). Fibrocartilaginous embolization to the spinal cord: serial MR imaging monitoring and pathologic study. *American Journal of Neuroradiology*.

[7] Jellema K., Canta L. R., Tijssen C. C., van Rooij W. J., Koudstaal P. J., van Gijn J. (2003). Spinal dural arteriovenous fistulas: clinical features in 80 patients. *Journal of Neurology, Neurosurgery and Psychiatry*.

[8] Fischer S., Kruger M., McRae K., Merchant N., Tsao M. S., Keshavjee S. (2001). Giant bronchial carcinoid tumors: a multidisciplinary approach. *The Annals of Thoracic Surgery*.

[9] Van Hilten J. J., Haan J., Wintzen A. R. (1989). Cerebral infarction in hereditary spherocytosis. *Stroke*.

[10] Becton D. L., Kletzel M., Arnold W. C., Berry D. H. (1988). Thrombotic thrombocytopenic purpura in an asplenic patient with hereditary spherocytosis: failure of plasmapheresis, antiplatelet therapy, and corticosteroids. *American Journal of Pediatric Hematology/Oncology*.

[11] McGrew W., Avant G. R. (1984). Hereditary spherocytosis and portal vein thrombosis. *Journal of Clinical Gastroenterology*.

[12] Sparwasser C., Danz B., Thon W. F. (1988). Segmental unilateral priapism—a case report. *Der Urologe*.

[13] De Backer A. I., Zachee P., Vanschoubroeck I. J., Mortele K. J., Ros P. R., Kockx M. M. (2002). Extramedullary paraspinal hematopoiesis in hereditary spherocytosis. *JBR-BTR*.

[14] Hayag-Barin J. E., Smith R. E., Tucker F. C. (1998). Hereditary spherocytosis, thrombocytosis, and chronic pulmonary emboli: a case report and review of the literature. *American Journal of Hematology*.

[15] Stewart G. W., Amess J. A. L., Eber S. W. (1996). Thrombo-embolic disease after splenectomy for hereditary stomatocytosis. *British Journal of Haematology*.

[16] Scholz K. H., Herrmann C., Tebbe U., Chemnitius J. M., Helmchen U., Kreuzer H. (1993). Myocardial infarction in young patients with Hodgkin's disease—potential pathogenic role of radiotherapy, chemotherapy, and splenectomy. *The Clinical Investigator*.

[17] Tai Y. T., Yu Y. L., Lau C. P., Fong P. C. (1993). Myocardial infarction complicating postsplenectomy thrombocytosis, with early left ventricular mural thrombus formation and cerebral embolism—a case report. *Angiology*.

[18] Schilling R. F., Gangnon R. E., Traver M. I. (2008). Delayed adverse vascular events after splenectomy in hereditary spherocytosis. *Journal of Thrombosis and Haemostasis*.

[19] Robinette C. D., Fraumeni J. F. (1977). Splenectomy and subsequent mortality in veterans of the 1939–45 war. *The Lancet*.

[20] Tso S. C., Chan T. K., Todd D. (1982). Venous thrombosis in haemoglobin H disease after splenectomy. *Australian and New Zealand Journal of Medicine*.

[21] Broe P. J., Conley C. L., Cameron J. L. (1981). Thrombosis of the portal vein following splenectomy for myeloid metaplasia. *Surgery Gynecology & Obstetrics*.

[22] Macchia P., Massei F., Nardi M., Favre C., Brunori E., Barba V. (1990). Thalassemia intermedia and recurrent priapism following splenectomy. *Haematologica*.

[23] Hirsh J., Dacie J. V. (1966). Persistent post-splenectomy thrombocytosis and thrombo-embolism: a consequence of continuing anaemia. *British Journal of Haematology*.

[24] Gordon D. H., Schaffner D., Bennett J. M., Schwartz S. I. (1978). Postsplenectomy thrombocytosis: its association with mesenteric, portal, and/or renal vein thrombosis in patients with myeloproliferative disorders. *Archives of Surgery*.

[25] Rodeghiero F., Ruggeri M. (2012). Short- and long-term risks of splenectomy for benign haematological disorders: should we revisit the indications?. *British Journal of Haematology*.

[26] Crary S. E., Buchanan G. R. (2009). Vascular complications after splenectomy for hematologic disorders. *Blood*.

[27] Westerman M. P. (1975). Hypocholesterolaemia and anaemia. *British Journal of Haematology*.

[28] Schilling R. F. (1997). Spherocytosis, splenectomy, strokes, and heart attacks. *The Lancet*.

[29] Goldschmidt N., Spectre G., Brill A. (2008). Increased platelet adhesion under flow conditions is induced by both thalassemic platelets and red blood cells. *Thrombosis and Haemostasis*.

[30] de Jong K., Larkin S. K., Eber S., Franck P. F. H., Roelofsen B., Kuypers F. A. (1999). Hereditary spherocytosis and elliptocytosis erythrocytes show a normal transbilayer phospholipid distribution. *Blood*.

